# Limited Pollen Dispersal Contributes to Population Genetic Structure but Not Local Adaptation in *Quercus oleoides* Forests of Costa Rica

**DOI:** 10.1371/journal.pone.0138783

**Published:** 2015-09-25

**Authors:** Nicholas John Deacon, Jeannine Cavender-Bares

**Affiliations:** 1 Department of Ecology, Evolution and Behavior, University of Minnesota, St. Paul, Minnesota, United States of America; 2 Plant Biological Sciences Graduate Program, University of Minnesota, St. Paul, Minnesota, United States of America; National Cheng-Kung University, TAIWAN

## Abstract

**Background:**

*Quercus oleoides* Cham. and Schlect., tropical live oak, is a species of conservation importance in its southern range limit of northwestern Costa Rica. It occurs in high-density stands across a fragmented landscape spanning a contrasting elevation and precipitation gradient. We examined genetic diversity and spatial genetic structure in this geographically isolated and genetically distinct population. We characterized population genetic diversity at 11 nuclear microsatellite loci in 260 individuals from 13 sites. We monitored flowering time at 10 sites, and characterized the local environment in order to compare observed spatial genetic structure to hypotheses of isolation-by-distance and isolation-by-environment. Finally, we quantified pollen dispersal distances and tested for local adaptation through a reciprocal transplant experiment in order to experimentally address these hypotheses.

**Results:**

High genetic diversity is maintained in the population and the genetic variation is significantly structured among sampled sites. We identified 5 distinct genetic clusters and average pollen dispersal predominately occurred over short distances. Differences among sites in flowering phenology and environmental factors, however, were not strictly associated with genetic differentiation. Growth and survival of upland and lowland progeny in their native and foreign environments was expected to exhibit evidence of local adaptation due to the more extreme dry season in the lowlands. Seedlings planted in the lowland garden experienced much higher mortality than seedlings in the upland garden, but we did not identify evidence for local adaptation.

**Conclusion:**

Overall, this study indicates that the Costa Rican *Q*. *oleoides* population has a rich population genetic history. Despite environmental heterogeneity and habitat fragmentation, isolation-by-distance and isolation-by-environment alone do not explain spatial genetic structure. These results add to studies of genetic structure by examining a common, tropical tree over multiple habitats and provide information for managers of a successional forest in a protected area.

## Introduction

Genetic diversity and structure have long been recognized as critical to the maintenance of viable and resilient populations [1] and as the engine for speciation [2, 3]. Genetic diversity is the raw material that determines the capacity of populations to respond to change and represents an important buffer against extinction [4]. The loss of genetic diversity within and among populations is currently thought to be occurring at a rapid rate relative to species extinctions [5–7]. In an era when most biological systems are human dominated, understanding the spatial arrangement of genetic variability and the factors that drive spatially structured genetic diversity are critical to conservation.

The factors that drive spatially structured genetic diversity are the subject of long-standing inquiries in population and landscape genetics [8–10]. Genetic structure among plant populations can arise due to processes that influence gene flow patterns such as founder events, inbreeding, isolation by distance, isolation by environment, and complex historical processes that include repeated population expansion, contraction and migration [11]. Isolation-by-distance (IBD) [12] is observed when gene flow between populations declines with increasing spatial distance allowing subsequent genetic drift to cause genetic differentiation among populations. Declining gene flow with distance is expected based on observed patterns of pollen and seed dispersal, given that most pollen deposition, pollination events and seedling establishment occur near the parent plant [13]. Spatially structured genetic diversity can also arise through isolation-by-environment (IBE), whereby gene flow among populations inhabiting dissimilar abiotic or biotic environments is limited as a consequence of adaptive differentiation among populations and selection against dispersing individuals [14]. Differences in the timing of reproduction among populations represent another important mechanism that can limit gene flow and lead to spatially structured genetic variation. Inter-site differences in the timing and availability of water may influence flowering time and reproductive phenology [15] because flower production requires significant hydraulic support to develop and sustain desiccation-sensitive tissues [16]. For example, divergent selection acts on flowering time in populations that differ in soil moisture [17] and the timing of snowmelt in alpine species [18, 19]. Flowering time asynchrony resulting from environmental heterogeneity has been increasingly recognized as a potential driver of diversification and population structure [20–22].

Highly polymorphic neutral genetic markers are widely used to assess associations between environmental variation and population genetic structure [23, 24] and such associations have been used as indicators of the influence of abiotic factors (elevation, aspect, soil type, temperature, precipitation, etc.) on the spatial distribution of genetic variation across many taxa [25–27]. Local adaptation and adaptive differentiation are known to be important drivers of spatial genetic structure [28–30] but require experimental approaches to detect [31, 32]. Recent studies have attempted to quantify the relative roles of IBD and IBE on spatial genetic structure by correlative comparisons to various climatic or environmental features [33, 34]. Few studies, however, have integrated neutral-marker population structure observations with explicit tests for local adaptation.

Three conditions are thought to be necessary for local adaptation to occur: 1) high genetic variation exists within the population; 2) individuals within a population are distributed over varying environmental conditions; and 3) gene flow is extremely limited or absent among areas of the range [35, 36]. Initial observations suggest that these conditions are likely to be met in the lone lowland tropical oak species of northwestern Costa Rica, *Quercus oleoides*. The large population size until recent land-use change likely resulted in high genetic diversity within the population [37]. Considerable heterogeneity exists in climate, with highly seasonal precipitation at low elevations and a much less severe dry season at high elevations [38]. Gene flow may also be limited among sites, as has been shown in other oak species [39]. Seed movement is known to be limited to very short distances due to the poor dispersal behavior displayed by the primary mammal disperser, *Dasyprocta punctata* (Central American Agouti) [40]. Therefore, wind-dispersed pollen movement is likely a more important mechanism of gene flow in this population. Pollen dispersal may also be limited, however, given that *Q*. *oleoides* displays considerable variability in flowering phenology [37]. The distribution of the population across climatic and elevation gradients coupled with the potential for limited gene flow sets up the possibility that selection across this gradient has led to local adaptation.

*Quercus oleoides*, tropical live oak, is an important study system in terms of conservation. It provides important ecosystem services and is considered to be critical to restoration of Central American tropical dry forests [41]. *Quercus oleoides* is the dominant primary producer in the seasonally dry tropical forest (SDTF) of Costa Rica, a highly threatened tropical ecosystem heavily cleared and fragmented for cattle and crops over the last century [42, 43]. The species forms monodominant stands capable of persisting in poor quality soils [44, 45]. The live oak ecosystem is distinct in its evergreen canopy in the SDTF where many species are drought deciduous, thus providing unique habitats that support biodiversity at other trophic levels [37, 41]. Diversity and function of this unique ecosystem thus depends on the population persistence of *Q*. *oleoides*. Its range extends primarily along the Atlantic coast to northern Mexico, but the genetically distinct Costa Rican population is separated from the rest of the range by the Nicaraguan Depression and is on the Pacific slope [46, 47].

The purpose of this study is to examine the genetic diversity and structure of this important tropical tree species by 1) identifying how much standing genetic variability exists in this population; 2) characterizing population genetic structure; and 3) examining possible drivers of landscape scale genetic structure by quantifying pollen dispersal distances and testing for locally adapted genotypes. Specifically, we test three possible mechanisms that could contribute to landscape scale genetic structure, including isolation by distance, isolation by time (via asynchronous flowering) and isolation by environment. Finally, using a reciprocal transplant experiment, we tested the hypothesis that progeny from opposite ends of the primary climatic and elevation gradient within the population would be locally adapted.

## Materials and Methods

### Research Site and Sampling

**Fig 1:**
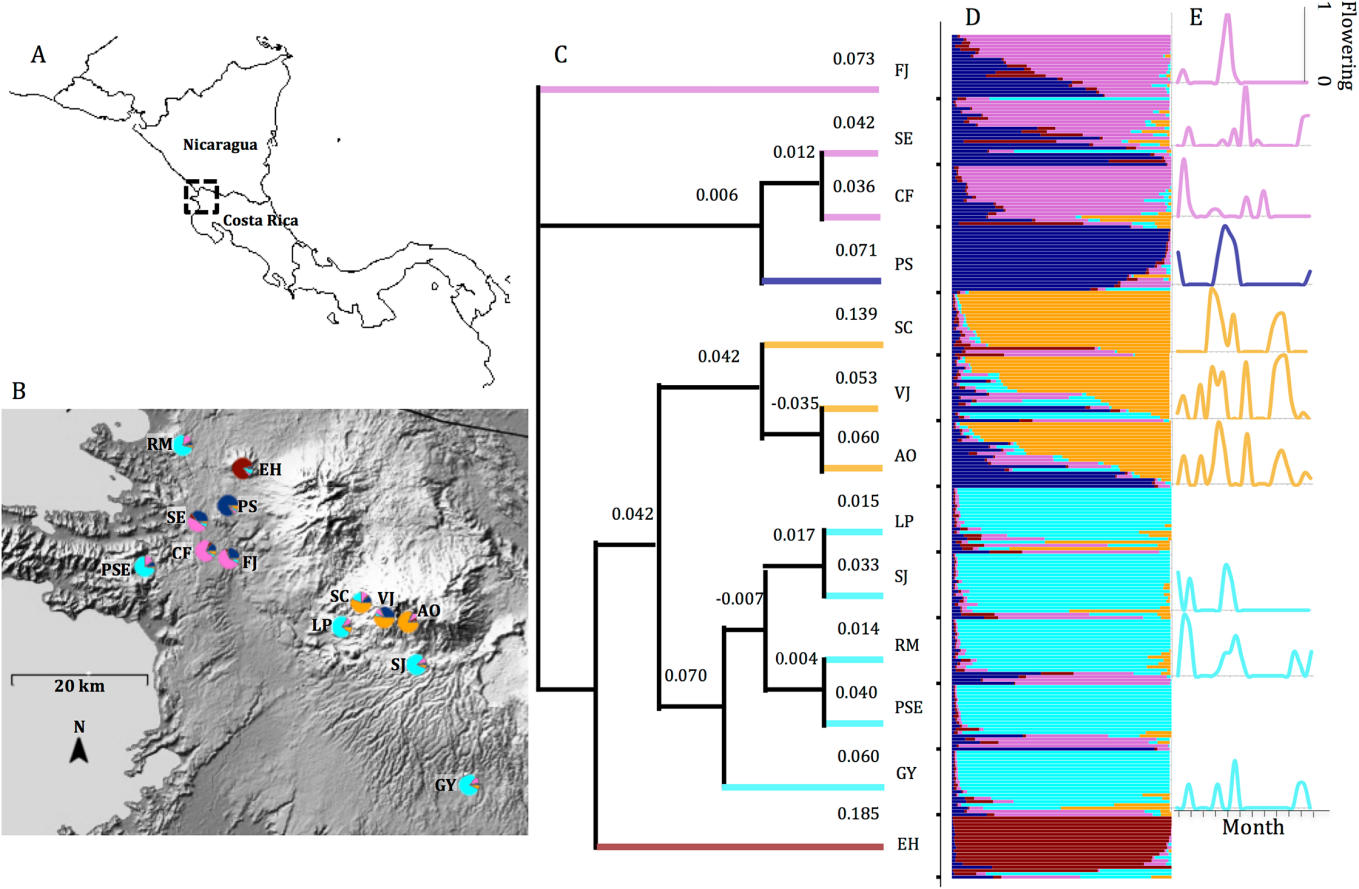
Study area and results of microsatellite data. A)Area of study in Costa Rica enclosed in dashed box. B) Close up of area in dashed box with sampling sites plotted and labeled (see Table 1 for GPS coordinates). Pie colors indicate proportion of individuals from each site assigned to each of 5 STRUCTURE clusters (see Results). Image cropped from: http://photojournal.jpl.nasa.gov/catalog/PIA03364. C) UPGMA tree using Nei’s genetic distances among 13 sampling sites. Numbers indicate branch length. D) STRUCTURE clustering results for K = 5. Horizontal bars indicate the proportion of individuals from 13 sites assigned to the 5 populations identified by STRUCTURE based on 11 nuclear microsatellites. The y-axis represents the 257 individuals in the analysis. E) Staminate flower observations at 10 of 13 sites. Size of peak represents the proportion of observed trees with male flowers present.

This study was conducted in the tropical dry forests of the 120,000-hectare Área de Conservación Guanacaste (ACG) in northwestern Costa Rica (Fig 1A). Prior to the establishment of this conservation region in 1971, decades of fragmentation occurred that transformed thousands of acres of dry forest into cattle pastures. Over the past 40 years parcels of land have been added to the ACG, resulting in a mosaic of live oak forest and pasture that is no longer under agricultural management.

We collected leaf tissue from 20 individuals at each of 13 sites (Fig 1B). These sites represent the geographic and environmental range of *Q*. *oleoides* in Costa Rica (permit number: ACG-PI-026-2006, Guanacaste Conservation Area, National System of Conservation Areas, Ministry of Environment and Energy). Duplicate sampling of vegetative clones was avoided by sampling non-neighboring trees separated by at least 25 meters. Each sampled tree was marked and its location was recorded (S1 Table). Young leaves were preferentially collected and stored in a -20°C freezer for up to two weeks until the beginning of the DNA extraction process. DNA was extracted using Qiagen DNeasy Plant Mini Kits in lab facilities at Costa Rica’s Santa Rosa National Park. The extracted DNA was quantified using an UV/Vis spectrophotometer at the University of Minnesota and diluted with distilled water to a concentration of approximately 10 ng/mL for polymerase chain reaction (PCR) amplification.

### Microsatellite Data Collection

Nine of the eleven microsatellites employed (ZAG15, ZAG110, ZAG 9, ZAG 1/2, ZAG 1/5, ZAG 102, ZAG 36, ZAG 16, ZAG 46) were developed for *Q*. *petraea* by Steinkeller *et al*. [48] The other two (ZAG 30 and ZAG 11) were developed by Kampfer *et al*. [49] for *Q*. *robur*. A multiplex PCR approach was taken where each PCR reaction included 3 or 4 primer pairs. Each 10 μl PCR reaction consisted of 1.0 μl of the fluorescently labeled forward primer and 1.0 μl of the reverse (10 μM each), 5.0 μl Qiagen Master Mix, 1 μl (~10 ng) DNA, and sterilized water. The thermal cycles for this reaction were: 94°C for 15 min, followed by 35 cycles of 30 s at 94°C, 30 s at 50°C and 60 s at 72°C. A final extension step of 10 min at 72°C was added after the last cycle. An ABI 377 Automated Sequencer at the University of Minnesota’s Advanced Genetics Analysis Center was used to measure the length of the fluorescently labeled PCR product. We used the program GenoProfiler to characterize alleles by the length of the amplified fragments [50]. In the original publications of these microsatellite markers, the authors found them all to fit neutral expectations. We confirmed this using an Ewens-Watterson test in the program PopGene [51].

### Genetic diversity and spatial structure

We calculated the percentage of polymorphic loci, average number of alleles per locus, observed and expected heterozygosities [52], and a fixation index averaged over all loci for all individuals at each site. Genetic differentiation between sampling sites was determined by the infinite allele model F_ST_ and analysis of molecular variance (AMOVA) [53]. Among population differentiation was also quantified by R_ST_ using a stepwise-mutation model. Differences between F_ST_ and R_ST_ can indicate the relative importance of drift and mutation in causing the observed differentiation [54]. The degree of genetic similarity between sites was based on Nei’s genetic distance [55].

The number of genetically distinct clusters was estimated using a Bayesian approach in which a Markov Chain Monte Carlo method clustered individuals to minimize Hardy-Weinberg disequilibrium and linkage disequilibrium (STRUCTURE [56]). This method does not assume a particular mutation model and probabilistically assigns individuals into K populations, or multiple populations if there is admixture. Ten runs were performed for each K (putative number of distinct genetic clusters) and the range of possible K’s tested was from 1 to 18. The USEPOPINFO flag was set to 0, commanding the program not to take the sampling location into account in assigning clusters. Runs were performed with a burn-in length of 50,000 and a MCMC of 1,000,000. The correct K was selected by observing the highest log likelihood values (ln Pr[X|K]) averaged over the 10 runs. Evanno *et al*. [57] detected the correct K by comparing the magnitude of the change in log likelihood value (delta K) because of problems with increasing log likelihood scores with increasing K’s.

STRUCTURE results were compared to a similar clustering analysis performed by the program InStruct [58] where Hardy-Weinberg equilibrium within loci is not assumed. InStruct estimates an optimal K using a deviance information criterion (DIC) and a posterior likelihood for each K is also generated.

### Environmental Data Collection

We described the environment of each site using elevation, the Holdridge life zone classification system, and a climate classification specific to Costa Rica. The Holdridge system defines life zones based on annual precipitation and biotemperature (∑ mean monthly temperature >0°C /12) [59]. Because the seasonal fluctuation in precipitation is obscured by annual data, we included the climate classification from Herrera that quantifies the effect of the dry season [60]. It comprises an aridity index (degree of water deficit below water need) and a hydric index (amount of water available for plants) (Table 1, S2 Table). These measures are commonly used in biogeography studies because of strong association with vegetation [61].

**Table 1:** Environmental characterization for 13 sampling sites based on elevation, Holdridge (1969) Life Zones, and Herrera (1986) climate classification for Costa Rica.

Site	Elevation (ft)	Life Zone^Ω^	Climate type^∂^	Lat	Long
FJ*	675	mf-P Basal	B1	10.86	-85.57
SE*	900	mf-P Basal	B1	10.91	-85.61
CF*	900	mf-P Basal	B1	10.87	-85.56
SC*	2600	wf-P	E8	10.78	-85.35
VJ*	2700	mf-T Prem	E8	10.78	-85.37
AO*	2800	wf-P	E8	10.79	-85.36
PS	1000	mf-P Basal	B4	10.93	-85.57
EH^	1000	mf-P Basal	B4	10.98	-85.55
LP^	1700	mf-T	C4	10.77	-85.42
SJ	2000	mf-T Prem	D4	10.72	-85.32
GY	475	df-T	A1	10.56	-85.25
PSE^	1000	mf-P Basal	B1	10.85	-85.68
RM	800	mf-P Basal	B1	11.01	-85.63

*sites where detailed environmental data were collected (average daily high and low temperature, average daily high/low relative humidity, and monthly soil moisture (S1 Fig).

*^sites not included in monthly phenology monitoring*.

^*Ω*^ *Holdridge classification (Part A S2 Table).*

^*∂*^ *Herrera classification (Part B S2 Table).*

At three low elevation and three high elevation sites we collected detailed soil moisture, daily photon flux density, (PFD) temperature (T) and relative humidity (Rh) data which show the large environmental gradient in a more quantitative manner (Table 1 and S1 Fig)

At 10 of the 13 sites we made phenological observations on one focal tree and its ten nearest neighbors. We recorded the presence of male and female flowers once per month for two years. It was not possible to visit the other three sites (EH, LP, PSE) every month due to poor accessibility.

### Pollen dispersal distance estimation

Pollen dispersal distances were estimated through molecular characterization of individuals using eight microsatellite loci developed for other species in the genus *Quercus* by Kampfer (ZAG 30 in *Q*. *robur*) and Steinkeller (ZAG 110, ZAG 15, ZAG 9, ZAG ½, ZAG1/5, ZAG102, ZAG 35 in *Q*. *petraea*) [49, 62]. We employed two computational methods for estimating pollen dispersal distance based on microsatellites: 1) the TwoGener (two generation) method of Smouse and others [63–67] and 2) the paternity exclusion methods found in the program FaMoz by Gerber *et al*. [68]. In the TwoGener approach, mother trees are effectively acting as pollen traps and the heterogeneity of their sampled pollen pools is compared. It requires genotyping fewer offspring than parentage methods, but relatedness among individuals and highly structured genetic variation can affect results. Parentage methods are advantageous in that distances of fathers can be accurately established with thorough sampling, but they often yield only minimal dispersal distances because much pollen arrives from outside the specified radius. By employing both the TwoGener and parentage methods, a more informative picture of the shape of the pollen dispersal decay curve can be drawn and long distance gene flow can be better inferred [69].

For the TwoGener analysis, we genotyped leaf tissue from 22 germinated acorns from each of ten variably spaced mother trees (S2A Fig) Maternal and progeny genotypes were characterized by assaying the eight microsatellite markers described above. The mother trees were chosen from throughout the landscape so that multiple distance comparisons can be made. A genetic distance matrix was constructed to estimate intergametic genetic distances of pollen, and an analysis of molecular variance (AMOVA) was used to determine variation in pollen donors within one tree and between pairs of trees. The fraction of total variance accounted for by interfemale distance is estimated by ϕ_FT_ (analogous to F_ST_ for male gametes) using the spatial heterogeneity in male gametes. ϕ_FT_ is inversely proportional to mean pollination distance and provides an estimate of effective pollination neighborhood size [66, 67]. The TwoGener procedure was implemented in GenAlEx [70]. The number of effective pollen donors (N_ep_) can also be derived from ϕ_FT_ in TwoGener [63]. We also used the Austerlitz and Smouse [67] approach to estimate dispersal distance using the bivariate normal distribution as recommended, but there is considerable debate about the proper distribution to use to model pollen dispersal [71].

For the paternity exclusion method 63 adult trees (over 20cm DBH) within a 50-meter radius of one central mother tree were genotyped. Leaf tissue from the mother tree and 29 progeny were also genotyped. We used the program FaMoz to calculate exclusion probabilities using likelihood methods to estimate the number of likely pollen donors from within the 50m radius [68]. The method uses the microsatellite allele frequencies to compare the likelihood ratio between all possible father-offspring pairs and the observed data. The higher the likelihood score for any particular parent-offspring pair, the more likely it is true. A threshold likelihood score was developed through simulating progeny by random association of gametes, and paternity was attributed to the pollen pool coming from outside the stand in observed likelihood scores fell below the threshold value.

### Mantel tests for correlation with genetic distance

We used Mantel tests to quantify the correlation between Nei’s genetic distance and the geographic distance among sites [72]. Spatial distance was obtained using GPS coordinates in the center of each sampling site. Significance was determined by comparing the observed correlation to random permutations of the data. Additionally, we calculated a multivariate spatial autocorrelation coefficient following Smouse and Peakall [73]. Spatial distance classes were chosen following the protocol recommended by the software program authors. All analyses were conducted using GenAlEx [70] and Arlequin [74].

Normalized Euclidean distances among the sites were calculated from elevation, life zone, and climate type to make a matrix of environmental dissimilarity using the program Primer [75]. Correlations between the genetic distance matrix and the environmental distance matrix were examined and significance was determined by comparing the observed correlation to random permutations of the data.

Similarity of flowering time among sampling sites was calculated according to Schoener’s index of similarity [76] to describe the proportion of trees that were flowering synchronously. Values were calculated using a Visual Basic program described in Cavender-Bares *et al*. [77].

Partial Mantel tests, where the correlation between two matrices is tested while removing the effect of a third matrix, were calculated in the program ZT to compare matrices while excluding spatial distance [78]. We compared distance matrices using Mantel tests because our data could be readily converted into measures of site similarity or dissimilarity. Spurious correlations have been detected in simulation studies that question the reliability of Mantel tests [79] and methods have been developed to address these concerns [80]

### Seedling and garden establishment and tests for local adaptation

Reciprocal transplant experiments are powerful means to detect local adaptation [32, 81] and are often performed on short-lived perennial or annual plants because fecundity is easily measured. This is not a required condition, however, because when individual fecundity cannot be measured in long-lived perennials and trees, traits relating to size or growth have been shown to be effective fitness surrogates [82–84].

We collected at least 50 acorns from 26 trees; thirteen maternal families from low elevation sites and thirteen maternal families from high elevation sites. Acorns were germinated and seedlings were grown in plastic nursery bags with a 1:1 mix of sand and peat. Acorn diameter and length were measured on a sample of seeds from a subset of the families to estimate acorn volume, which was then converted to mass based on a calibration curve derived from sampled measurements. The seedling nursery was covered with 70% shade cloth. Seedlings were well watered and grew for 2–3 months prior to transplantation. Two weeks before transplantation, we removed the shade cloth and reduced watering to allow for acclimation.

Two gardens were established in old pastures adjacent to current *Q*. *oleoides* stands. Existing pasture vegetation was cleared, fenced, and covered with plastic sheeting in order to prevent competition of other vegetation with the seedlings. The lowland garden (280 meters above sea level) was located near the seedling nursery in sector Santa Elena (10°55’12”N, 85°36’39”W; near site SE), and the upland garden (800 meters above sea level) was located near the entrance to Parque Nacional Rincón de la Vieja (10°46’23”N, 85°21’03”W; near site SC)(S2B Fig). The seedlings were arranged in a randomized block design over three blocks, and an equal number of seedlings per family were represented in each garden.

1668 seedlings were planted through the plastic sheet in October 2006 and censused at least twice per year over the next three years. After six months, the plastic sheeting was removed and the growth of pasture vegetation was maintained by periodic mowing. To evaluate the effects of source population, location and their interactions on overall fitness, we used the maximum-likelihood approach called Aster Models [83, 85] in the R package, “aster” (R Development Core Team). This method is an improvement over ANOVA methods because fitness components (for example survival and growth over multiple seasons and stages) are modeled with different statistical distributions and because it accounts for the dependence of fitness components expressed later in the life of the organism on those expressed earlier [83].

Survival at each time period, modeled as a Bernoulli distribution, and final leaf number, modeled as a Poisson distribution, were integrated for all analyses in Aster. Leaf number was chosen as a surrogate measure of fitness because it is has been shown to be correlated with biomass in a sister species, *Quercus virginiana* (R^2^ = 0.58, unpublished data[86]). We fit multiple models of increasing complexity to the integrated survival and final leaf number data and compared them with likelihood-ratio tests. We compared how a model (model 2) that included family fit the data compared to a simpler model of garden, block and source (model 1). We also compared the model that included family (model 2) to one that included a family x garden interaction (model 3). The test for local adaptation looks for a significantly better fit to the data for a model containing a source x garden interaction (model 4) compared to a model without that interaction (model 1). Local adaptation is inferred if fitness of the home source trees is higher in the home site relative to its fitness elsewhere.

## Results

### Genetic diversity and spatial structure

A total of 257 individuals were successfully assayed at 11 microsatellite loci. Allelic diversity was high with each locus containing between 6 and 17 alleles (Table 2). Each site averaged at least four alleles per locus and 91%- 100% of the loci were polymorphic per site. Average expected heterozygosity, H_e_, for all sites was 0.628.

**Table 2:** Genetic diversity estimates for each site averaged over 11 microsatellite loci. **N sample size, NA number of alleles, P percentage polymorphic loci, H**_**o**_ **observed heterozygosity, H**_**e**_ **expected heterozygosity, F fixation index. Loci (italics) and alleles per locus (parentheses): *ZAG 15* (7), *ZAG 30* (11), *ZAG 110* (6), *ZAG 9* (13), *ZAG 1/2* (8), *ZAG 1/5* (7), *ZAG 102* (17), *ZAG 36* (9), *ZAG 16* (10), *ZAG 46* (9), *ZAG 11* (6).**

Site	N	N_A_	%P	H_o_	H_e_	F
FJ	19.5	5.8	100%	0.639	0.619	-0.046
SE	19.7	5.4	100%	0.471	0.552	0.121
CF	18.8	5.2	100%	0.607	0.599	-0.019
SC	19.0	4.4	100%	0.608	0.562	-0.084
VJ	19.5	5.4	100%	0.634	0.621	-0.036
AO	19.8	5.2	100%	0.577	0.608	0.034
PS	19.7	4.4	100%	0.543	0.570	0.027
EH	18.7	4.0	100%	0.506	0.560	0.079
LP	20.0	4.3	91%	0.513	0.521	0.017
SJ	19.5	3.8	91%	0.553	0.512	-0.069
GY	20.0	4.7	100%	0.559	0.513	-0.03
PSE	19.4	4.4	91%	0.517	0.495	-0.016
RM	19.5	4.0	91%	0.520	0.487	-0.065

Genetic variation was significantly partitioned among the 13 sampled sites based on an AMOVA (F_ST_ = 0.102, p = 0.01 & R_ST_ = 0.15, p<0.001, Table 3). An UPGMA tree [87] based on Nei’s distances illustrates that there are 4 or 5 clusters of genetically similar sites (Fig 1C).

**Table 3:** Analysis of molecular variance (AMOVA) of 11 microsatellites using F_ST_ and R_ST_ for 13 sampling sites.

**Source**	**df**	**SS**	**MS**	**Est. Var.**	**%**
*Among Pops*	12	207.493	17.291	0.358	10%
*Within Pops*	501	1570.905	3.136	3.136	90%
*Total*	513	1778.399	20.427	3.494	
F_ST_ = 0.102, p = 0.01					
**Source**	**df**	**SS**	**MS**	**Est. Var.**	**%**
*Among Pops*	12	35254.116	2937.843	64.953	15%
*Within Pops*	501	185260.863	369.782	369.782	85%
*Total*	513	220514.979	3307.625	434.736	
R_ST_ = 0.15, p<0.001					

There are several accepted methods for interpreting the results from Bayesian clustering analyses [57]. We examined the clustering output from values of K that were considered likely by comparing their posterior probabilities from STRUCTURE as well as by the delta K method. The peak in the log likelihood score from STRUCTURE shows that the most likely K value is 5, and the delta K method also show a major peak at 5 (although 2 clusters had a slightly higher peak) The InStruct DIC score suggested an optimal K of 11, but the DIC scores and mean posterior likelihood did not differ significantly after peaking near K = 5 (S3 Fig). The output from K = 5 best captured the variability among sites and there was general agreement between Structure, InStruct, and the UPGMA tree (Fig 1D). Pie charts in Fig 1B are colored to indicate the assignment of individuals from each site to the 5 STRUCTURE clusters. S4 Fig shows comparisons of various K values between STRUCTURE and InStruct.

### Pollen dispersal distance

Genotyping of the individuals in both the TwoGener analysis and the paternity exclusion analysis yielded high diversity and 100% polymorphic loci. The number of alleles per locus ranged from 6 (ZAG ½) to 20 (ZAG 102). For the TwoGener analysis, we successfully genotyped and average of 20 offspring from 10 mothers. The inter-mother distances varied from less than one kilometer to over 60 km. The majority of the total variation in male gametes (87%) was found within mothers, but a statistically significant 13% of the total variation in male gametes occurred among mothers (ϕ_FT_ = 0.131 p<0.001, Table 4). Pairwise ϕ_FT_ values between mothers ranged from 0.04–0.23. On average only 3.8 pollen donors fathered the 22 offspring per mother. We used the default dispersal curve in TwoGener (bivariate normal) and our own density measurement (81 trees per hectare) to obtain an estimate of eight meters for the average effective pollen dispersal distance. In the paternity exclusion, 8 microsatellite markers were sufficient to exclude potential fathers with high probability (cumulative exclusion probability over 8 loci = 0.997), and there was very little chance any two individuals drawn at random from the population could not be differentiated (Probability of identity <0.001). The likelihood approach of FaMoz successfully excluded all but one potential father for 24 of the 29 genotyped offspring. Fathers for five of the 29 offspring were likely found outside of the 50-meter radius of the study stand, yielding an inflow rate of approximately 17%.

**Table 4:** AMOVA table from TwoGener analysis showing a statistically significant 13% of the total variation in paternal gametes occurs among mothers

Source	df	SS	MS	Est. Var.	%
Among Mothers	9	74.356	8.262	0.305	13%
Within Mothers	195	395.369	2.028	2.028	87%
Total	204	469.726		2.332	100%

### Mantel tests for correlation with genetic distance

**Fig 2:**
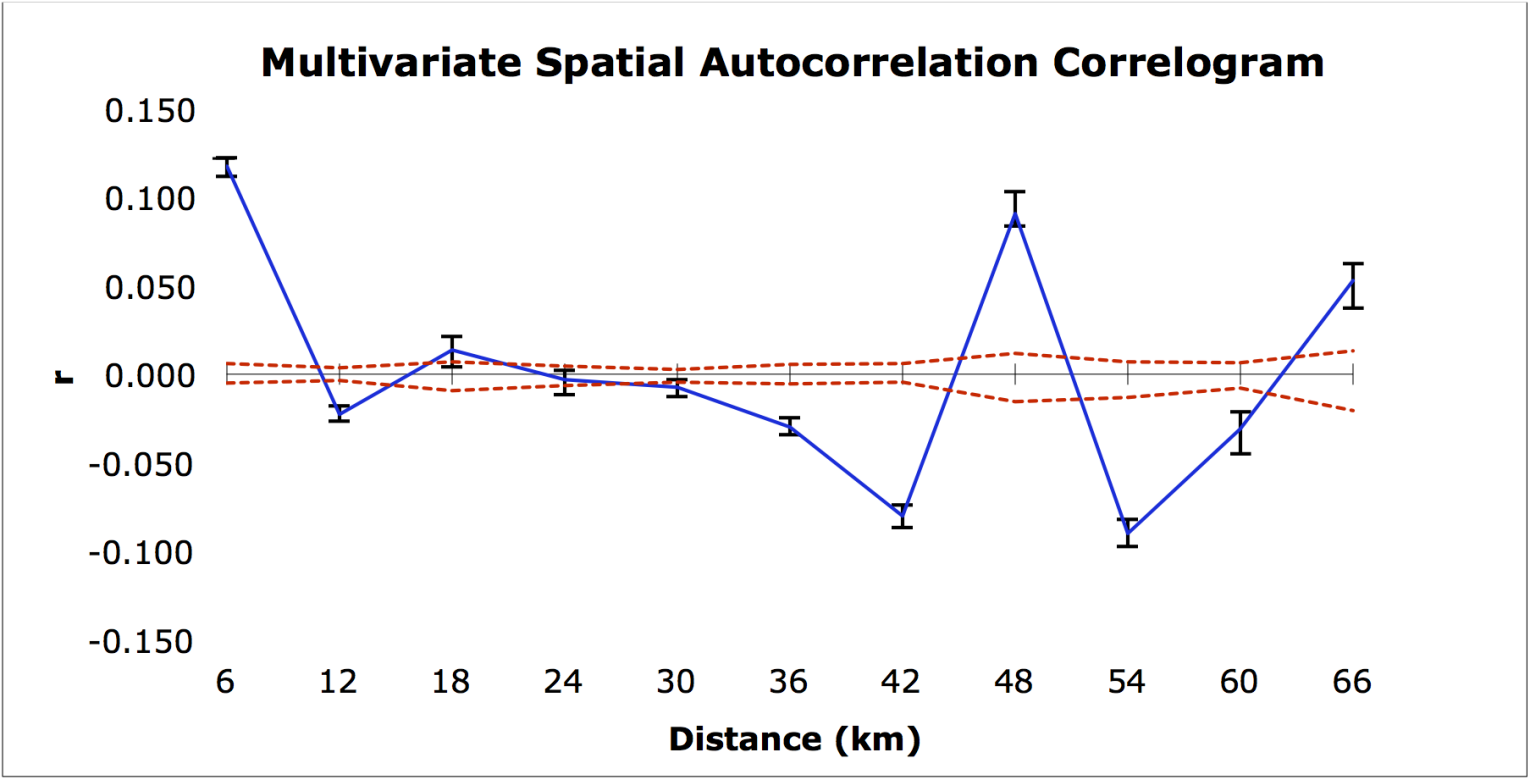
Correlogram from spatial autocorrelation analysis. Solid line indicates the autocorrelation coefficient, r (-1–1), between individuals at each distance class. Dashed lines are 95% confidence intervals based on 1000 random permutations of all individuals. Values above and below dashed lines indicate significant spatial structure. Error bars result from 1000 permutations of individuals within each distance class.

There was no significant correlation between the genetic distance matrix and the spatial distance matrix (r = 0.17, p = 0.14; Table 5). The multivariate spatial autocorrelation analysis illustrated that those individuals less than 6 km apart had non-random genetic similarities, as did those that were about 48 km and 66 km apart (Fig 2).

**Table 5:** Mantel tests for correlation between matrices. Partial Mantel test compares first two matrices over residuals of third matrix

Mantel Test	Pearson correlation coefficient	Pearson correlation coefficient: *no Zag15*
Genetic-Spatial	0.17	0.05
Genetic-Environmental	0.29*	0.12
Genetic-Phenologic	-0.08	-0.04
Environmental-Phenologic	-0.28*	
**Partial Mantel Test**		
Genetic-Environmental-Spatial	0.25*	0.11

1000 permutations

* = p<0.*05*.

The environmental distance matrix was significantly correlated with the full genetic distance matrix (r = 0.29, p = 0.017 all loci; Table 5). The Ewen’s-Watterson test for neutrality did not indicate non-neutral behavior for any of the loci, but a locus-by-locus analysis found that only ZAG15 had a significant correlation with the environmental distance matrix (r = 0.65, p = 0.001). When this locus was removed from the genetic distance matrix, the environmental distance matrix was no longer significantly correlated with the genetic distance matrix (r = 0.12, p = 0.16). A partial Mantel test between the genetic distance and environmental distance, partitioning out the effects of spatial distance, was significant when ZAG 15 was included (r = 0.25, p = 0.052; Table 5) but not when it was removed (r = 0.11, p = 0.21; Table 5).

Phenological observations provided evidence for a large amount of variability in staminate flowering time among the 10 observed sites (Fig 1E). Some sites flowered as many as seven months per year, whereas others only three. While we did find a significant correlation between environmental distance and flowering time overlap (r = -0.28, p<0.05), there was no significant correlation between genetic distance and flowering time overlap across the 10 sites (r = -0.08, p = 0.28 all loci, Table 5). All Mantel test matrices can be found in S3 Table.

### Test for locally adapted genotypes

**Fig 3:**
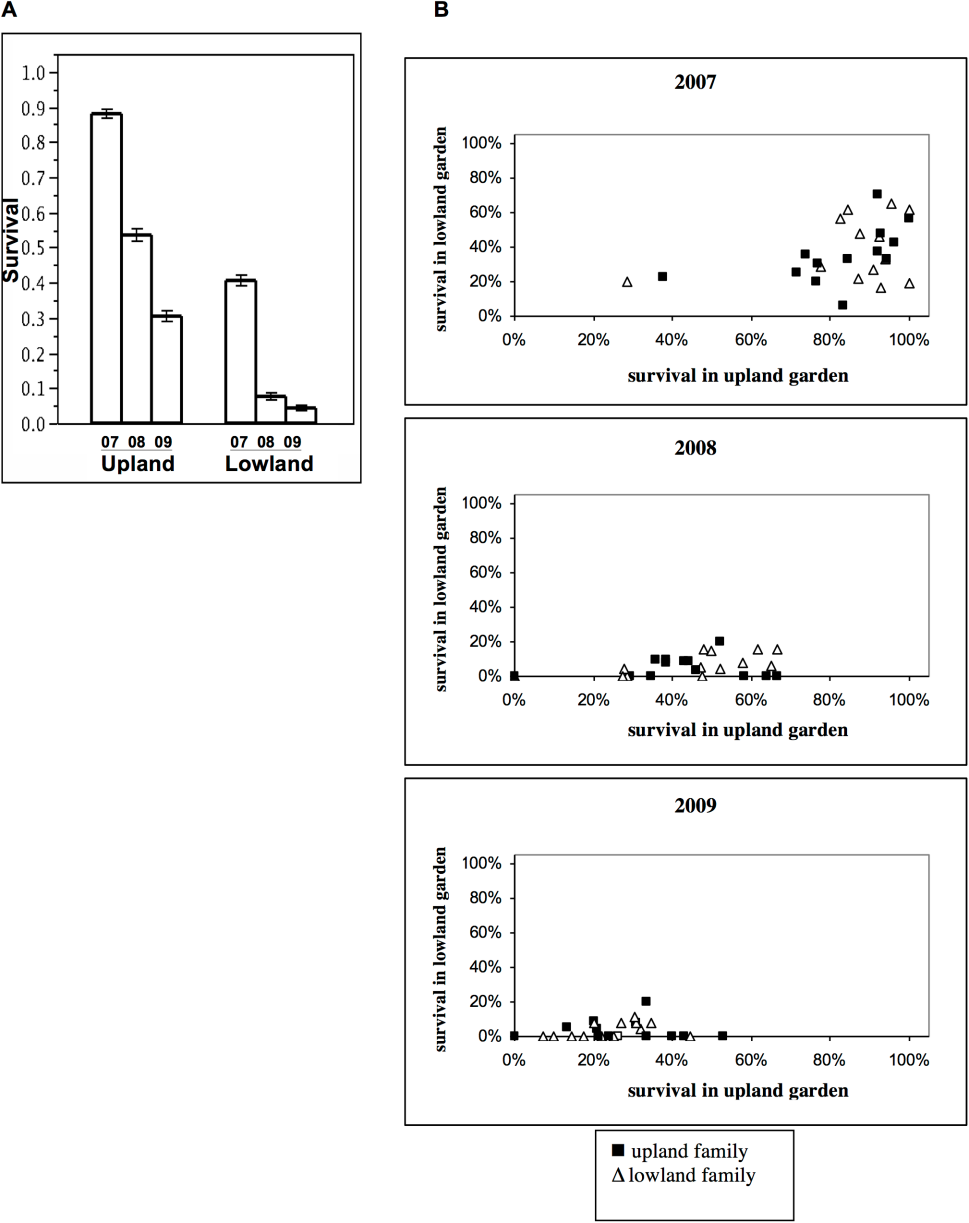
Average seedling survival in upland and lowland gardens. A) January 2007, January 2008, and January 2009. Error bars are standard errors. B) Upland families are solid shapes and lowland families are open.

Average survival was considerably reduced in the lowland garden compared to the upland garden, but mortality increased in each sampling period in both gardens (Fig 3A). Three years after initial planting, fewer than 40% of seedlings planted in the upland garden survived and fewer than 10% of seedlings planted in the lowland garden survived. Average family survival was greater in the upland garden for both upland and lowland populations (Fig 3B).

Acorn mass was highly variable among families (1.6 grams– 7.6 grams, S5 Fig). However, there was no evidence for a significant difference in the average acorn mass per population. In the Aster analyses, we found that a model containing family fit the data better than a model without the factor of family (Table 6). Also, a model containing a family x garden interaction fit the data better than without the interaction (Table 6). In the test for local adaptation, comparing the model containing a garden x source interaction to one without that interaction, the model with the interaction did not fit the data significantly better (Table 6).

**Table 6:** Likelihood ratio tests from Aster models comparing models of increasing complexity to test for significant factors in the model.

Model	Df	ModelDev	Df	Deviance	P(>|Chi|)
***1***	17	-87106			
***2***	62	-87269	45	163	>0.001*
***2***	62	-87269			
***3***	107	-87374	45	106	>0.001*
***1***	17	-87106			
***4***	19	-87110	2	4	0.1506

*Model 1 (garden*:*block*, *soure)*

*Model 2 (garden*:*block*, *source*, *family)*

*Model 3 (garden*:*block*, *source*, *family x garden)*

*Model 4 (garden*:*block*, *source*, *garden x source)*.

**Fig 4:**
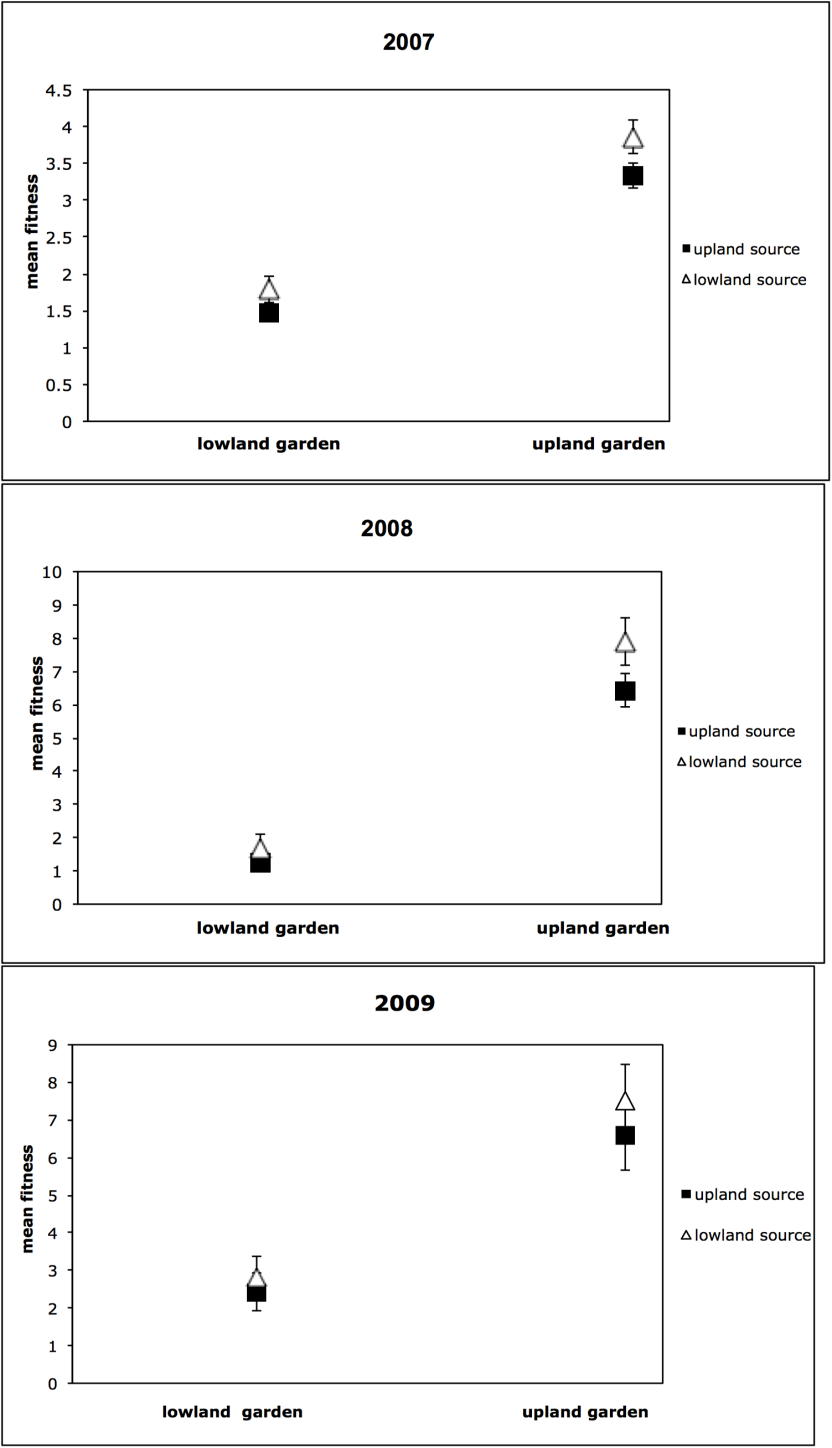
Plot of Aster fitness component (combined survival and number of leaves) averaged over three blocks for each source in the lowland and upland garden at three time intervals for the lowland and upland sources in the two gardens. A) Fitness comparison in January 2007, B) Fitness comparison in January 2008, C) Fitness comparison in January 2009.

A plot of the combined survival and leaf number fitness estimates shows that fitness was higher for both source trees in the upland garden (Fig 4). The mean for the lowland trees in the final year (7.16) was greater than for the upland trees (6.35) in the upland site as well as the lowland site (3.06 and 2.67 respectively). A comparison of Aster results over three sampling periods showed a trend toward a reduced difference in the mean fitness for each source in both gardens as the experiment progressed (Fig 4).

## Discussion

In the geographically isolated and ecologically important population of the Costa Rican lowland tropical oak, *Q*. *oleoides*, we show evidence for high genetic diversity, significant genetic structure among sites due to limited gene flow and short pollen dispersal distances. However, we find no evidence for locally adapted genotypes at extreme ends of the primary climatic and elevation gradient within the population.

### Genetic diversity and structure

The scattered fragments of this peripheral population contain high genetic diversity and significant population genetic structure. Genetic diversity in this population is quite high (H_e_ = 0.628 ± 0.52) suggesting that potential adaptive capacity to future environmental change has not been lost. The 13 sites had a statistically significant level of among-site genetic variation (Table 3). Differentiation among populations under a stepwise-mutation model, as measured by R_ST_, showed slightly higher values than F_ST_, indicating that geographic isolation might contribute to population differentiation [54]. This level of genetic subdivision in a relatively small area is rare for a wind-pollinated, long-lived tree species. Population genetic structure is largely absent in abundant and widespread temperate oak species of North America, for example [88–91]. A recent analysis of the southern range edge of another tropical oak species, however, showed similarly strong spatial genetic structure over a relatively small area that also appears to be primarily driven by geographic distance despite environmental heterogeneity there as well [92].

#### Examining evidence for isolation-by-distance and isolation-by-environment

In an attempt to explain the causes of significant population genetic structure, we examined mechanisms that could lead to the genetic isolation of sites allowing alteration of allele frequencies through genetic drift. We compared distance matrices using Mantel tests because our data could be readily converted into measures of site similarity or dissimilarity. There was no significant correlation between the genetic distance matrix and the spatial distance matrix (Table 5), however, isolation by distance appears to account for population genetic structure over short spatial scales in the spatial autocorrelation analysis (Fig 2). This process cannot explain how some physically distant populations are genetically more similar than some that are physically nearby. Fig 1B–1D shows the clustering of three lowland sites (SE, CF, and FJ) and three upland sites (SC, VJ, and AO), while the two sites, PS and EH, are predominantly unique clusters. The cluster identified by STRUCTURE, InStruct, and the UPGMA tree made up of individuals from five sites (LP, SJ, GY, PSE, RM) represents individuals from the entire North-South distribution in Costa Rica, but these sites occur all along the western periphery of the range. This result is also seen in the spatial autocorrelation where the observed r falls significantly above (6, 48, and 66 km) and below (12, 42, 54, 60 km) the dashed line, which represents the null hypothesis of no spatial genetic structure, indicating that spatial distance does not consistently predict genetic distance across spatial scales (Fig 2).

Environmental variation and flowering time variation do not provide a simple explanation for population genetic structure either. There was no significant genetic differentiation associated with the environment, with the exception of a single locus, ZAG15. This locus may be in linkage disequilibrium with a region of the genome that is affected by some or all of the factors in the environmental distance matrix. Other studies in the genus *Quercus* have found significant associations between microsatellite markers and genes coding for traits under selection [82, 93]. ZAG15 has been mapped to linkage group 9 near QTL’s for leaf shape [94] and bud burst [95], and a study in *Q*. *petraea* found an association between ZAG15 and altitude [96].

Nevertheless, the timing of the presence of male flowers is correlated with environmental variation, indicating that temperature, humidity, and/or soil moisture may influence the timing of flower production (Table 5). Despite asynchrony in staminate flowering time over the observation period, it was not significantly correlated with overall genetic distance (Table 5) over the timescale of the study. In the higher elevation sites, we observed staminate flowers on at least one tree in 22 of the 24 months that we made observations (Fig 1E). In contrast, at lower elevations staminate flowers were only observed during the wet season, indicating greater seasonality. The frequency of individuals flowering at any one time or in one particular year has an influence on whether or not phenological assortative mating could result [97]. A longer term study of reproductive phenology of both male and female flowers would provide a better mechanistic understanding of pollination relevant to the long lifespan and large number of reproductive years of the species [98].

### Infrequent long distance pollination

Evidence for very restricted pollen movement emerged from two different approaches for evaluating pollen dispersal distance. The TwoGener analysis showed an average effective dispersal distance of eight meters and the paternity analysis indicated that pollen donors for 83% of progeny likely come from within a 50-meter radius. Other studies that utilized both of these types of analyses have observed a discrepancy between the methods where the paternity method sometimes yields greater estimated distances [69], but our results seem to support one another.

The low number of effective pollen donors per mother, 3.8, is comparable to other *Quercus* species in disturbed landscapes (e.g. N_ep_ = 3.68 for *Q*. *lobata*, [99], N_ep_ = 5.1–6.1 for *Q*. *humboldtii* [100], and N_ep_ = 8.22 *Q*. *alba* [67]. Our estimated average pollen dispersal distance of 8m for *Q*. *oleoides* is on the low end in comparison with other *Quercus* species (17m for *Q*. *alba* and 65m for *Q*. *lobata*). The large difference in estimated pollen dispersal distance between *Q*. *lobata* and *Q oleoides* (65m and 8m) can be explained by the higher density of *Q*. *oleoides* (1.19/hectare and 81/hectare). Other wind dispersed species have been shown to similarly derive paternity from nearby individuals (*Fraxinus* [101] and *Populus* [102]). In all these studies, the possibility for rare long-range pollination is still present and much debate continues over appropriate shape for modeling the pollen dispersal curve [71]. The 17% inflow rate, or the proportion of genotyped progeny fathered by individuals outside the sampled radius, observed in the paternity exclusion study is less than those observed in other *Quercus* species as well (e.g. *Q*. *macrocarpa* = 0.57 [103].

### No evidence for local adaptation

We found no evidence that progeny from contrasting climatic and elevation sites were locally adapted. Given limits to gene flow across this gradient, we hypothesized that differences in the abiotic conditions of the lowland and upland environments might select against seedlings from the opposite environment. The results of our Aster analysis of the fitness of reciprocally transplanted progeny did not support this hypothesis, but the common garden results do corroborate results from the Mantel tests indicating little evidence for the hypothesis of isolation by environment. The model that included a source x garden interaction did not fit the data significantly better than a model without that interaction (Table 6). There was a large difference in overall survival and fitness between gardens, and we attribute this difference to the increased dry season severity of the lowland environment. The disparity could be exacerbated because seedlings were planted into an open environment rather than in the canopy understory.

The fitness of families from the lowland population was higher in the lowland environment (Fig 4). That relationship was maintained in the upland environment, suggesting that lowland populations have an advantage in both environments. A possible explanation for the consistently higher fitness of the lowland population is that the lowland populations have undergone selection for increased plasticity relative to the upland population, due to experiencing a more variable environment [104–106]. Increased plasticity resulting from high environmental heterogeneity has been shown to be correlated with greater genetic variability [30] but we did not observe significantly greater genetic diversity (H_e_) in the lowland population. Another possible interpretation for the consistently higher fitness of the lowland population is that unaccounted factors, such as greater allocation to below ground growth or the presence of maternal effects that caused lowland populations to have larger seed sizes. If families from the lowland population had larger overall seed sizes, then the higher leaf number in lowland individuals would not be a result of increased seedling performance [107]. In at least two other oak species, *Quercus suber* and *Quercus douglasii*, it has been shown that acorn size is larger in populations from drier habitats [82, 108]. However, there was no significant difference in the size of the acorns from different populations.

The higher mean fitness of the lowland population relative to the upland population in both environments decreased as time progressed and was no longer significant by the third year. The advantage by the lowland population may thus be ephemeral or dependent on momentary environmental conditions, disappearing by the time these long-lived trees reach reproductive maturity. Our lack of evidence for local adaptation may be dependent on the life stage we examined. We chose to focus on achieving the desired amount of replication by planting seedlings into the gardens. However, it is possible that by not planting seeds directly in the gardens we missed an important life history stage that could have revealed a signal of local adaptation [109].

Maternal family was a significant factor in the model, and there was a significant family x garden interaction. This suggests heritability of the fitness trait and therefore the potential to respond to natural selection. Our fitness trait is a composite of survival and leaf number and we observed wide variability among mean family survival (Fig 3). We also saw large among family differences in a subsample of seed masses, (S5 Fig) making it likely that the small seeded families suffered greater mortality, especially in the foreign garden, and this led to the significant family and family x garden effects. We hope to continue investigating the heritability of these traits with additional experiments designed explicitly for this purpose.

## Conclusion

Patterns of spatial genetic variation, and the role that landscape heterogeneity plays in shaping those patterns, continues to grow as an area of research interest [23]. We characterized population genetic structure and tested hypotheses of IBD and IBE in an attempt to explain the significant genetic structure in the Costa Rican population of tropical live oak, *Quercus oleoides*. Our results indicate that isolation by distance may be the dominant force shaping population genetic structure among some sites and at small distances, but environmental variation likely plays a role as well. Our methods did not allow us to precisely attribute proportions of total genetic variation to IBD or IBE, but other studies have found a dominant role of IBE in certain cases with IBD still playing a role [33, 34, 92]. Our study goes beyond other investigations of IBD and IBE that rely solely on genetic marker data and broad climatic associations to make inferences about process (e.g. [110]). We integrated marker and climatic data with measurements of gene flow via effective pollen dispersal and an explicit test for locally adapted genotypes to gain insights into the biological processes underlying patterns attributed to IBD and IBE.

The fact that we do not see local adaptation given that conditions were met for it to be present is surprising; and although detecting local adaptation in early seedling stages of long-lived trees is challenging, the significant family x garden interaction suggests the potential for future adaptation [111]. Despite limits to geneflow, sufficient pollen dispersal among these sites may still be present to dominate over divergent selection [112]. Molecular markers do not necessarily indicate heritable variation in adaptive traits [113], and neutral genetic variation does not reflect adaptive variation. Examples exist in other *Quercus* species where morphology remains consistent despite high variation in molecular markers [114]. Our molecular evidence reveals the predominance of short distance pollen movement, but also indicates potential for long-range dispersal over the fragmented landscape.

Differential gene exchange due to asymmetrical pollen or seed dispersal, chance associations caused by genetic drift or founder effects, selection on genomic regions near the surveyed microsatellite markers [25] and complex historical processes associated with repeated expansion, contraction, and migration of populations during dynamic periods of climate change [115] and volcanic activity [46] have all been shown to play a part in driving the complex patterns of population genetic structure. Our data do not support just one explanation for the observed population genetic structure. As methods for measuring the frequency and relative importance of long distance pollen dispersal continue to be refined and improved, so too will our understanding of the reproductive history and future of wind pollinated trees in disturbed habitats. Phenomena such as masting [116] and anisotropy [117] have not been reported here and future studies could investigate the role of these, as well as a more careful evaluation of pistillate flower production and receptivity, in shaping population genetic structure.

Similar to other studies attempting to uncover the processes that create population genetic structure, our data do not support just one explanation. Because nearly the entire extent of this population now occurs within a conservation region, however, we have shown that the remnant oak stands are likely to harbor the diversity necessary for the recovery of part of the tropical dry forest ecosystem. The genetic diversity of this relatively small population (H_e_ = 0.63) nearly matches that of the entirety of it’s range (H_e_ = 0.77) while also being significantly subdivided geographically such that it should more correctly be thought of as 5 distinct populations. The Costa Rican *Q*. *oleoides* have already been shown to be genetically unique from the rest of the range [118] and a keystone tree species in the tropical dry forest [37]. Our results strengthen the conservation mission of the ACG by highlighting the importance of protecting habitat heterogeneity for the preservation of genetic diversity.

## Supporting Information

S1 Fig Principal coordinates analysis using environmental data. Temp, Rh, light, and soil moisture collected from three upland sites (SC = sendero caballo, VJ = valle jabely, AO = agave oaks) and three lowland sites (FJ = finca jenny, CF = corta fuego, SE = santa elena). Arrows illustrate eigenvectors for the variables. PCA axis 1 is significantly correlated with elevation (R^2^ = 83.2%, p = 0.011).
(PDF)

S2 Fig Pollen dispersal and common garden map. A) Map of TwoGener mother trees, paternity analysis tree, and phenology monitoring sites. B) Map of common garden locations and areas where maternal family seed sources were collected.
(PDF)

S3 Fig Evaluation of K from Structure and InStruct. A) Log likelihood probability (Ln PD) estimates. B) delta K values for the number of distinct ancestral admixture groups. C) Mean likelihood estimates for K values from InStruct. D) Deviance information criterion (DIC) scores for K values from InStruct.
(PDF)

S4 Fig Comparison of Bayesian clustering analyses using InStruct and Structure for several K values and all individuals from the 13 sampling sites. K = 5 was supported by the Evanno et al. (2005) delta K method for Structure results. K = 11 was considered the “optimal K” from Instruct
(PDF)

S5 Fig Family mean seed mass. Comparison of mean seed mass from a subsample of upland and lowland maternal families.
(PDF)

S1 File Sampled sites microsatellite data. (CSV)

S2 File Paternity analysis microsatellite data. (CSV)

S3 File TwoGener analysis microsatellite data. (CSV)

S4 File Common garden data. (CSV)

S1 Table Individual tree size and location data. (PDF)

S2 Table Key to life zone (A) and climate classification (B) information in Table 1. (PDF)

S3 Table Distance matrices for Mantel tests. A) Genetic distance matrix. B) Spatial distance matrix. C) Environmental distance matrix. D) Flowering time similarity matrix.
(PDF)
