# Supplementary figures and images for: Limited Pollen Dispersal Contributes to Population Genetic Structure but Not Local Adaptation in Quercus oleoides Forests of Costa Rica
Source: PLoS One. 2015 Sep 25;10(9):e0138783. doi: 10.1371/journal.pone.0138783 (PMC4583504; doi:10.1371/journal.pone.0138783)

Environmental data PCA

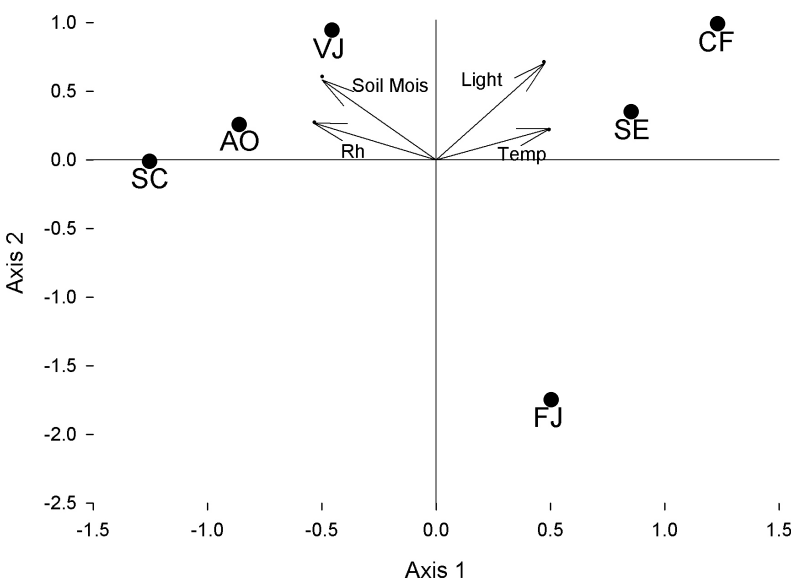

Supplement: S1 Fig — Temp, Rh, light, and soil moisture collected from three upland sites (SC = sendero caballo, VJ = valle jabely, AO = agave oaks) and three lowland sites (FJ = finca jenny, CF = corta fuego, SE = santa elena). Arrows illustrate eigenvectors for the variables. PCA axis 1 is significantly correlated with elevation (R2 = 83.2%, p = 0.011). (PDF) [file pone.0138783.s001.pdf]

**S5 Fig. Mean family seed mass from a subset of seeds from lowland and upland families.**

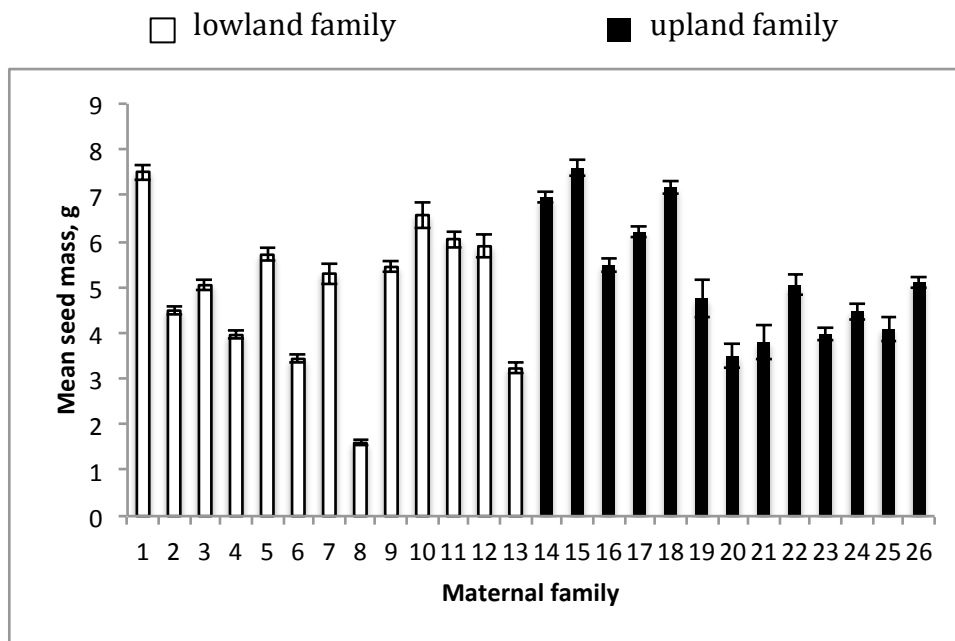

Supplement: S5 Fig — Comparison of mean seed mass from a subsample of upland and lowland maternal families. (PDF) [file pone.0138783.s005.pdf]
