# Supplementary material for: Limited Pollen Dispersal Contributes to Population Genetic Structure but Not Local Adaptation in Quercus oleoides Forests of Costa Rica
Source: PLoS One. 2015 Sep 25;10(9):e0138783. doi: 10.1371/journal.pone.0138783 (PMC4583504; doi:10.1371/journal.pone.0138783)
Supplement: S2 Fig — A) Map of TwoGener mother trees, paternity analysis tree, and phenology monitoring sites. B) Map of common garden locations and areas where maternal family seed sources were collected. (PDF) [file pone.0138783.s002.pdf]

S2 Fig. Map of pollen dispersal and common garden sites. A) Sites where mothers and progeny were collected for TwoGener analysis (stars), paternity analysis (circle), and flowering phenology monitoring (squares). GPS points for mother trees on right of map. B) Sites of common gardens (stars) and area from where upland and lowland progeny were collected (dotted circle). GPS points for mother trees on right of map.

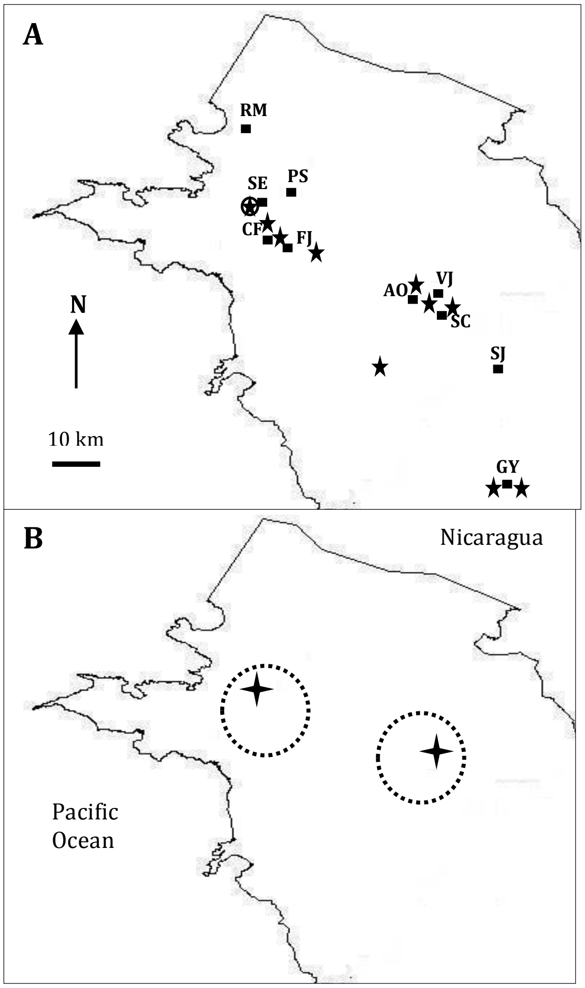

Lat.	Long.	Mother Tree
10.78	-85.35	M1
10.78	-85.36	M3
10.54	-85.32	M4
10.54	-85.34	M11
10.64	-85.45	M21
10.87	-85.58	M26
10.87	-85.60	M27
10.92	-85.62	M30
10.79	-85.36	M45
10.87	-85.60	M54

Lat.	Long.	Mother	pop.
10.85	-85.58	M24	lowland
10.85	-85.57	M25	lowland
10.87	-85.58	M26	lowland
10.87	-85.60	M27	lowland
10.84	-85.60	M28	lowland
10.92	-85.61	M29	lowland
10.92	-85.62	M30	lowland
10.87	-85.58	M33	lowland
10.71	-85.35	M34	lowland
10.92	-85.61	M49	lowland
10.92	-85.61	M50	lowland
10.92	-85.61	M52	lowland
10.87	-85.60	M54	lowland
10.78	-85.35	M1	upland
10.78	-85.37	M2	upland
10.78	-85.36	M3	upland
10.78	-85.37	M35	upland
10.78	-85.36	M36	upland
10.78	-85.35	M37	upland
10.79	-85.36	M40	upland
10.78	-85.37	M41	upland
10.78	-85.35	M44	upland
10.79	-85.36	M45	upland
10.78	-85.36	M46	upland
10.78	-85.37	M47	upland
10.77	-85.43	M48	upland
