# Supplementary material for: Limited Pollen Dispersal Contributes to Population Genetic Structure but Not Local Adaptation in Quercus oleoides Forests of Costa Rica
Source: PLoS One. 2015 Sep 25;10(9):e0138783. doi: 10.1371/journal.pone.0138783 (PMC4583504; doi:10.1371/journal.pone.0138783)

S3 Fig. Evaluation of K from Structure and InStruct (**A**) Log likelihood probability (Ln PD) estimates (**B**) delta K values for the number of distinct ancestral admixture groups. **(C)** Mean liklihood estimates for K values from InStruct. **(D)** Deviance information criterion (DIC) scores for K values from InStruct.

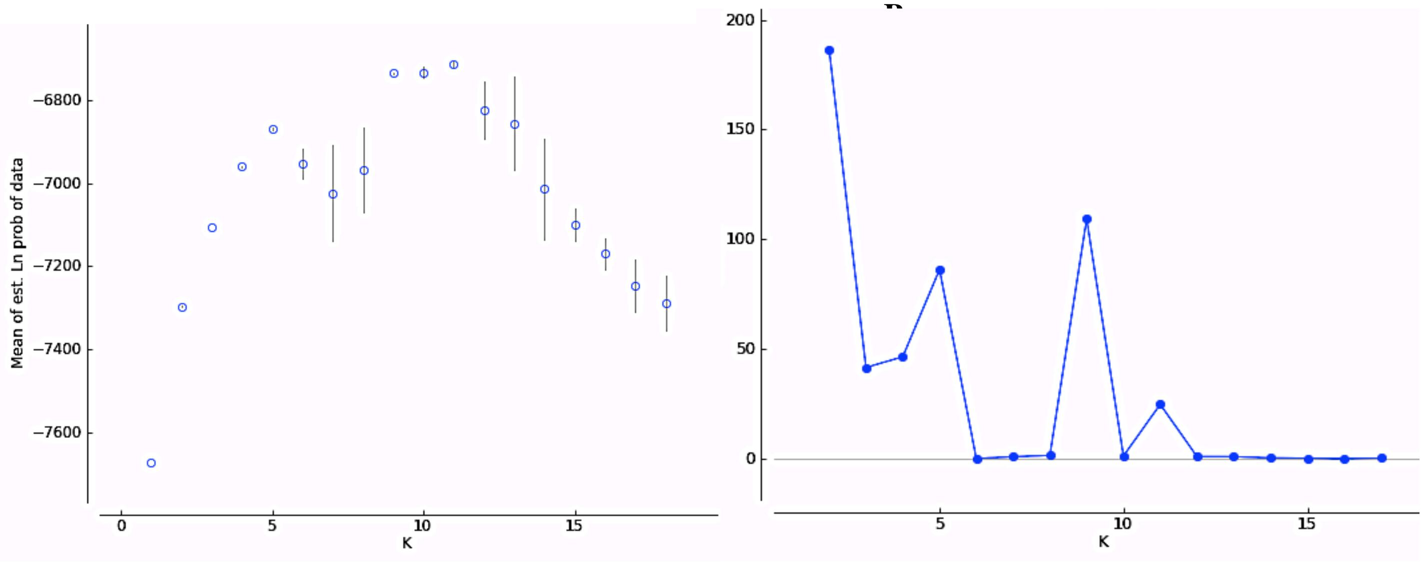

C

C **D**

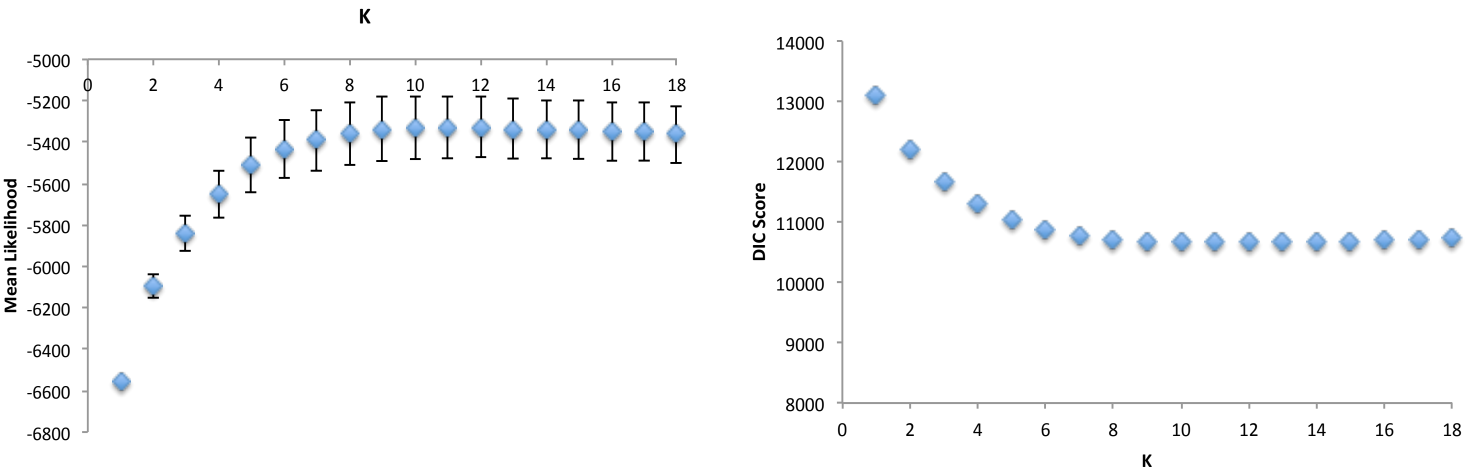

Supplement: S3 Fig — A) Log likelihood probability (Ln PD) estimates. B) delta K values for the number of distinct ancestral admixture groups. C) Mean likelihood estimates for K values from InStruct. D) Deviance information criterion (DIC) scores for K values from InStruct. (PDF) [file pone.0138783.s003.pdf]
