# Supplementary material for: Limited Pollen Dispersal Contributes to Population Genetic Structure but Not Local Adaptation in Quercus oleoides Forests of Costa Rica
Source: PLoS One. 2015 Sep 25;10(9):e0138783. doi: 10.1371/journal.pone.0138783 (PMC4583504; doi:10.1371/journal.pone.0138783)

S4 Fig. Comparison of Bayesian clustering analyses using InStruct and Structure for several K values and all individuals from the 13 sampling sites. K=5 was supported by the Evanno et al. (2005) delta K method for Structure results. K = 11 was considered the “optimal K” from Instruct

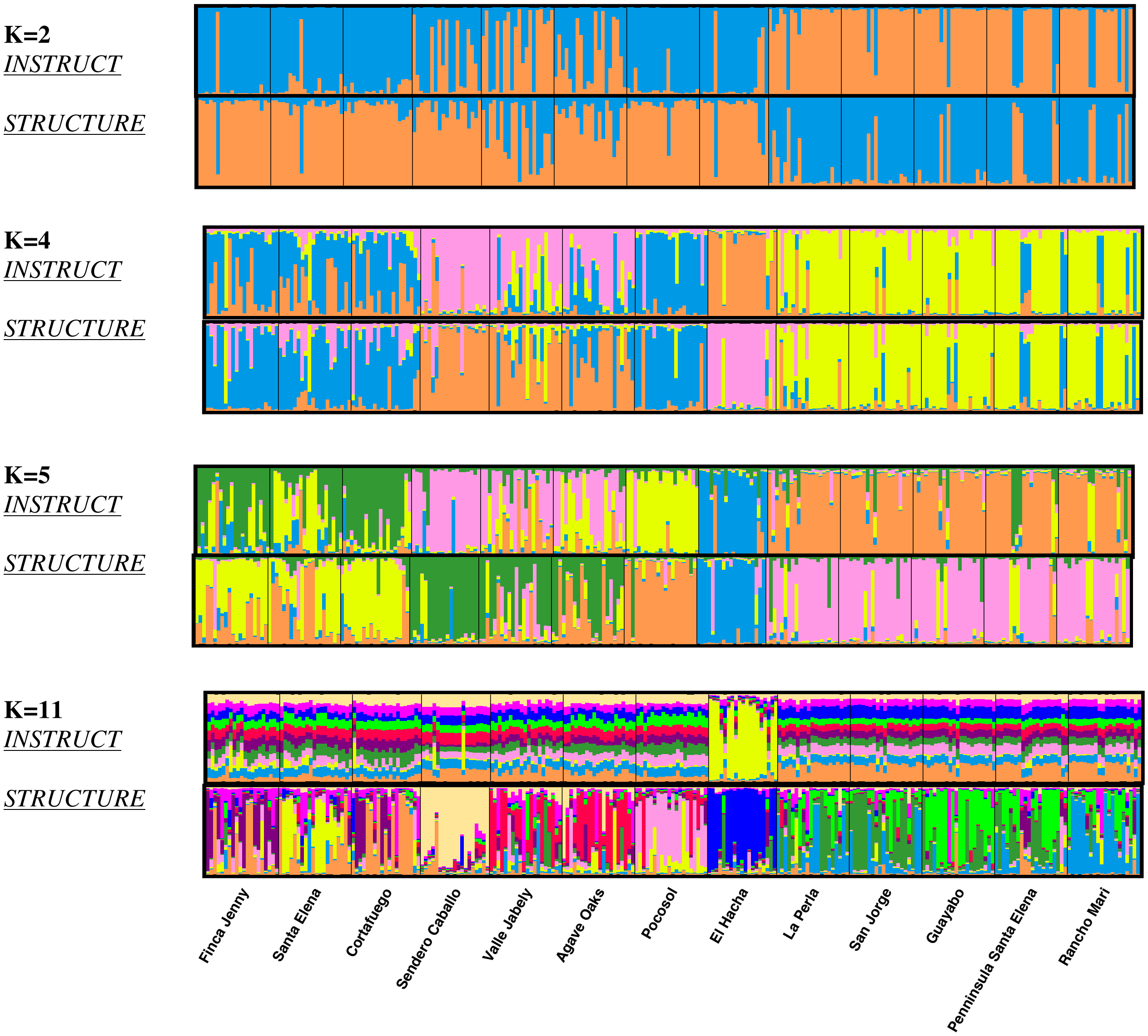

Supplement: S4 Fig — K = 5 was supported by the Evanno et al. (2005) delta K method for Structure results. K = 11 was considered the “optimal K” from Instruct (PDF) [file pone.0138783.s004.pdf]
