# Supplementary material for: Limited Pollen Dispersal Contributes to Population Genetic Structure but Not Local Adaptation in Quercus oleoides Forests of Costa Rica
Source: PLoS One. 2015 Sep 25;10(9):e0138783. doi: 10.1371/journal.pone.0138783 (PMC4583504; doi:10.1371/journal.pone.0138783)
Supplement: S1 Table — (PDF) [file pone.0138783.s010.pdf]

Appendix 1

Tree and seedling location and size (DBH in cm for adult trees only. All seedlings are less than 20cm tall)

SITE	TREE	DBH	LAT.	LONG.
CF	A1	52	10.8702	-85.5981
CF	A2	80	10.8707	-85.5983
CF	A3	56 & 70	10.8704	-85.5984
CF	A4	49	10.8705	-85.5987
CF	A5	61	10.8700	-85.5986
CF	A6	58	10.8700	-85.5986
CF	A7	58	10.8701	-85.5982
CF	A8	50	10.8700	-85.5984
CF	A9	62	10.8697	-85.5989
CF	A10	25.5 & 70	10.8700	-85.5984
CF	S1		10.8706	-85.5982
CF	S2		10.8705	-85.5986
CF	S3		10.8703	-85.5988
CF	S4		10.8700	-85.5986
CF	S5		10.8700	-85.5984
CF	S6		10.8702	-85.5984
CF	S7		10.8702	-85.5982
CF	S8		10.8701	-85.5983
CF	S9		10.8699	-85.5985
CF	S10		10.8701	-85.5980
FJ	A1	61	10.8624	-85.5756
FJ	A2	52	10.8620	-85.5756
FJ	A3	80	10.8620	-85.5754
FJ	A4	57	10.8622	-85.5755
FJ	A5	59	10.8623	-85.5754
FJ	A6	64	10.8620	-85.5752
FJ	A7	56	10.8618	-85.5751
FJ	A8	57	10.8624	-85.5750
FJ	A9	43 & 50	10.8627	-85.5752
FJ	A10	83	10.8624	-85.5749
FJ	S1		10.8622	-85.5756
FJ	S2		10.8621	-85.5754
FJ	S3		10.8622	-85.5755
FJ	S4		10.8623	-85.5752
FJ	S5		10.8620	-85.5752
FJ	S6		10.8618	-85.5748
FJ	S7		10.8621	-85.5748
FJ	S8		10.8625	-85.5751
FJ	S9		10.8628	-85.5751
FJ	S10		10.8621	-85.5751
SE	A1	65 & 24	10.9190	-85.6124
SE	A2	46	10.9190	-85.6128
SE	A3	50	10.9189	-85.6129
SE	A4	53	10.9187	-85.6130
SE	A5	38 & 51	10.9183	-85.6131
SE	A6	79	10.9182	-85.6129
SE	A7	26 & 33 & 34	10.9180	-85.6127
SE	A8	65	10.9185	-85.6128
SE	A9	40 & 27	10.9179	-85.6132
SE	A10		10.9180	-85.6132

SITE	TREE	DBH	LAT.	LONG.
SE	S1		10.9189	-85.6128
SE	S2		10.9186	-85.6130
SE	S3		10.9185	-85.6130
SE	S4		10.9184	-85.6130
SE	S5		10.9183	-85.6129
SE	S6		10.9185	-85.6125
SE	S7		10.9187	-85.6127
SE	S8		10.9187	-85.6124
SE	S9		10.9178	-85.6134
SE	S10		10.9177	-85.6134
SC	A1	80	10.7766	-85.3558
SC	A2	90	10.7770	-85.3553
SC	A3	75	10.7765	-85.3552
SC	A4	65	10.7764	-85.3554
SC	A5	75	10.7764	-85.3554
SC	A6	55	10.7764	-85.3554
SC	A7	70	10.7764	-85.3555
SC	A8	60	10.7764	-85.3555
SC	A9	80	10.7770	-85.3554
SC	A10			
SC	S1		10.7767	-85.3556
SC	S2		10.7768	-85.3555
SC	S3		10.7770	-85.3555
SC	S4		10.7770	-85.3552
SC	S5		10.7769	-85.3553
SC	S6		10.7768	-85.3550
SC	S7		10.7766	-85.3552
SC	S8		10.7766	-85.3552
SC	S9		10.7764	-85.3553
SC	S10		10.7765	-85.3553
VJ	A1	70	10.7794	-85.3676
VJ	A2	65	10.7795	-85.3676
VJ	A3	60	10.7795	-85.3676
VJ	A4	55	10.7795	-85.3677
VJ	A5	70	10.7794	-85.3677
VJ	A6	100	10.7794	-85.3678
VJ	A7	55	10.7795	-85.3679
VJ	A8	70	10.7795	-85.3680
VJ	A9	75	10.7795	-85.3681
VJ	A10	55	10.7799	-85.3691
VJ	S1		10.7794	-85.3681
VJ	S2		10.7793	-85.3681
VJ	S3		10.7793	-85.3679
VJ	S4		10.7793	-85.3677
VJ	S5		10.7794	-85.3677
VJ	S6		10.7796	-85.3676
VJ	S7		10.7795	-85.3677
VJ	S8		10.7796	-85.3677
VJ	S9		10.7796	-85.3676
VJ	S10		10.7797	-85.3691

SITE	TREE	DBH	LAT.	LONG.
AO	A1	65	10.7816	-85.3628
AO	A2	50	10.7816	-85.3629
AO	A3	60	10.7816	-85.3630
AO	A4	80	10.7817	-85.3635
AO	A5	75	10.7814	-85.3632
AO	A6	70	10.7821	-85.3632
AO	A7	65	10.7818	-85.3635
AO	A8	70	10.7819	-85.3634
AO	A9	90	10.7821	-85.3633
AO	A10	108	10.7820	-85.3634
AO	S1		10.7816	-85.3631
AO	S2		10.7812	-85.3631
AO	S3		10.7818	-85.3633
AO	S4		10.7816	-85.3633
AO	S5		10.7818	-85.3635
AO	S6		10.7820	-85.3633
AO	S7		10.7824	-85.3632
AO	S8		10.7823	-85.3634
AO	S9		10.7822	-85.3633
AO	S10		10.7818	-85.3634
PS	A1	71	10.9329	-85.5727
PS	A2	50	10.9326	-85.5726
PS	A3	105	10.9325	-85.5726
PS	A4	42 & 42	10.9324	-85.5726
PS	A5	62	10.9323	-85.5727
PS	A6	55	10.9321	-85.5727
PS	A7	87	10.9323	-85.5728
PS	A8	74	10.9324	-85.5727
PS	A9	24 & 30 & 36	10.9329	-85.5731
PS	L0142	34 & 34 & 35	10.9330	-85.5728
PS	S1		10.9324	-85.5728
PS	S2		10.9324	-85.5726
PS	S3		10.9325	-85.5726
PS	S4		10.9328	-85.5729
PS	S5		10.9331	-85.5730
PS	S6		10.9330	-85.5728
PS	S7		10.9328	-85.5726
PS	S8		10.9326	-85.5725
PS	S9		10.9325	-85.5725
PS	S10		10.9325	-85.5724
EH	A1	54	10.9835	-85.5535
EH	A2	100	10.9835	-85.5541
EH	A3	75	10.9836	-85.5543
EH	A4	180 & 200	10.9838	-85.5545
EH	A5	90	10.9841	-85.5544
EH	A6	120	10.9842	-85.5543
EH	A7	65	10.9844	-85.5544
EH	A8			
EH	A9	55	10.9838	-85.5552
EH	A10	65	10.9835	-85.5534

SITE	TREE	DBH	LAT.	LONG.
EH	S1		10.9835	-85.5535
EH	S2		10.9836	-85.5536
EH	S3		10.9836	-85.5540
EH	S4		10.9841	-85.5545
EH	S5		10.9838	-85.5547
EH	S6		10.9838	-85.5550
EH	S7		10.9838	-85.5550
EH	S8		10.9839	-85.5555
EH	S9		10.9838	-85.5556
EH	S10		10.9836	-85.5535
LP	A1	75	10.7688	-85.4256
LP	A2	65	10.7685	-85.4256
LP	A3	75	10.7685	-85.4253
LP	A4	65	10.7685	-85.4253
LP	A5	130	10.7685	-85.4249
LP	A6	90	10.7685	-85.4250
LP	A7	25 & 50	10.7684	-85.4249
LP	A8	85	10.7685	-85.4248
LP	A9	65	10.7684	-85.4258
LP	A10	120	10.7687	-85.4259
LP	S1		10.7686	-85.4256
LP	S2		10.7685	-85.4255
LP	S3		10.7686	-85.4254
LP	S4		10.7685	-85.4253
LP	S5		10.7685	-85.4252
LP	S6		10.7685	-85.4252
LP	S7		10.7684	-85.4250
LP	S8		10.7683	-85.4250
LP	S9		10.7686	-85.4248
LP	S10		10.7688	-85.4257
SJ	A1	50	10.7261	-85.3179
SJ	A2	75	10.7262	-85.3179
SJ	A3	80	10.7263	-85.3179
SJ	A4	120	10.7264	-85.3179
SJ	A5	65	10.7265	-85.3179
SJ	A6	50	10.7266	-85.3179
SJ	A7	50	10.7267	-85.3179
SJ	A8	50	10.7268	-85.3179
SJ	A9	50	10.7269	-85.3179
SJ	A10		10.7259	-85.3179
SJ	S1		10.7261	-85.3180
SJ	S2		10.7262	-85.3180
SJ	S3		10.7263	-85.3180
SJ	S4		10.7264	-85.3180
SJ	S5		10.7265	-85.3180
SJ	S6		10.7266	-85.3180
SJ	S7		10.7267	-85.3180
SJ	S8		10.7268	-85.3180
SJ	S9		10.7269	-85.3180
SJ	S10		10.7259	-85.3180

SITE	TREE	DBH	LAT.	LONG.
GY	A1	50	10.5639	-85.2542
GY	A2	45	10.5630	-85.2542
GY	A3	75	10.5631	-85.2542
GY	A4	100	10.5632	-85.2542
GY	A5	50	10.5633	-85.2542
GY	A6	70	10.5634	-85.2542
GY	A7	100	10.5635	-85.2542
GY	A8	40	10.5636	-85.2542
GY	A9	50	10.5637	-85.2542
GY	A10	76 & 40 & 23	10.5639	-85.2542
GY	S1		10.5646	-85.2538
GY	S2		10.5642	-85.2538
GY	S3		10.5643	-85.2538
GY	S4		10.5644	-85.2538
GY	S5		10.5645	-85.2538
GY	S6		10.5646	-85.2538
GY	S7		10.5647	-85.2538
GY	S8		10.5649	-85.2538
GY	S9		10.5650	-85.2538
GY	S10		10.5648	-85.2538
PSE	A1	90	10.8520	-85.6844
PSE	A2	100	10.8523	-85.6846
PSE	A3	75	10.8528	-85.6843
PSE	A4	60	10.8531	-85.6847
PSE	A5	46 & 40	10.8524	-85.6850
PSE	A6	91	10.8522	-85.6854
PSE	A7	80	10.8529	-85.6858
PSE	A8	96 & 17 & 13	10.8521	-85.6841
PSE	A9	73	10.8503	-85.6859
PSE	A10		10.8521	-85.6841
PSE	S1		10.8520	-85.6844
PSE	S2		10.8520	-85.6845
PSE	S3		10.8524	-85.6845
PSE	S4		10.8528	-85.6844
PSE	S5		10.8530	-85.6847
PSE	S6		10.8520	-85.6853
PSE	S7		10.8521	-85.6858
PSE	S8		10.8524	-85.6860
PSE	S9		10.8523	-85.6839
PSE	S10		10.8504	-85.6860
RM	A1	58	11.0136	-85.6376
RM	A2	62	11.0136	-85.6379
RM	A3	65	11.0136	-85.6382
RM	A4	47	11.0136	-85.6379
RM	A5	48	11.0134	-85.6369
RM	A6	35 & 35	11.0133	-85.6368
RM	A7	46	11.0133	-85.6371
RM	A8	32 & 25	11.0134	-85.6372
RM	A9	60.5	11.0137	-85.6380
RM	A10	15 & 13 & 19	11.0138	-85.6381
RM	S1		11.0136	-85.6378
RM	S2		11.0135	-85.6379
RM	S3		11.0136	-85.6381
RM	S4		11.0136	-85.6379
RM	S5		11.0134	-85.6370
RM	S6		11.0133	-85.6369
RM	S7		11.0134	-85.6369
RM	S8		11.0133	-85.6368
RM	S9		11.0148	-85.6350
RM	S10		11.0136	-85.6381
