# Supplementary material for: Limited Pollen Dispersal Contributes to Population Genetic Structure but Not Local Adaptation in Quercus oleoides Forests of Costa Rica
Source: PLoS One. 2015 Sep 25;10(9):e0138783. doi: 10.1371/journal.pone.0138783 (PMC4583504; doi:10.1371/journal.pone.0138783)
Supplement: S2 Table — (PDF) [file pone.0138783.s011.pdf]

S2 Table. Key to Life Zone and Climate Type classifications from Table 1.

[Ω]Life Zone

df-T	Tropical Dry Forest
mf-P Basal	Premontane Moist Forest Basal Transition
mf-T	Tropical Moist Forest
mf-T Prem	Tropical Moist Forest Premontane Transition
wf-P	Premontane Wet Forest

Holdridge classification from Tosi (1969).

[∂]Climate Type

Classification	Average Annual Precipitation (mm)	Average Annual Temperature (°C)	Annual Potential Evapotrans-piration(mm)	†Aridity Index (%)	‡Hydric Index (%)
A1	1300-1710	>27	>1710	>20	-33.3-0
B1	1710-2050	23-27	>1710	>20	0-20
B4	1565-2052	21-26	1565-1710	>20	0-20
C4	1900-2400	21-26	1565-1710	>20	20-40
D4	2200-2800	21-25	1565-1710	>20	40-60
E8	2300-2800	18-24	1420-1565	10-20	60-80

†*aridity index: 0-10 = 0-35 days of water deficit; 10-20 = 35-70 days of water deficit; >20 = >70 and <150 days of water deficit.*

‡*hydric index = ((average annual precipitation/annual potential evapotranspiration)-1) * 100.*

classification and data from Herrera (1986).
