# Supplementary material for: Limited Pollen Dispersal Contributes to Population Genetic Structure but Not Local Adaptation in Quercus oleoides Forests of Costa Rica
Source: PLoS One. 2015 Sep 25;10(9):e0138783. doi: 10.1371/journal.pone.0138783 (PMC4583504; doi:10.1371/journal.pone.0138783)
Supplement: S3 Table — A) Genetic distance matrix. B) Spatial distance matrix. C) Environmental distance matrix. D) Flowering time similarity matrix. (PDF) [file pone.0138783.s012.pdf]

## S3 Table. Distance matrices from Mantel tests

Population genetic distance matrix (Nei's genetic distance)

| | FJ | SE | CF | SC | VJ | AO | PS | EH | LP | SJ | GY | PSE | RM |
|---|---|---|---|---|---|---|---|---|---|---|---|---|---|
| **FJ** | 0.000 | | | | | | | | | | | | |
| **SE** | 0.154 | 0.000 | | | | | | | | | | | |
| **CF** | 0.108 | 0.078 | 0.000 | | | | | | | | | | |
| **SC** | 0.287 | 0.310 | 0.253 | 0.000 | | | | | | | | | |
| **VJ** | 0.188 | 0.198 | 0.182 | 0.184 | 0.000 | | | | | | | | |
| **AO** | 0.197 | 0.172 | 0.167 | 0.137 | 0.113 | 0.000 | | | | | | | |
| **PS** | 0.150 | 0.105 | 0.140 | 0.292 | 0.188 | 0.138 | 0.000 | | | | | | |
| **EH** | 0.185 | 0.261 | 0.286 | 0.362 | 0.316 | 0.326 | 0.290 | 0.000 | | | | | |
| **LP** | 0.235 | 0.177 | 0.163 | 0.281 | 0.167 | 0.176 | 0.254 | 0.287 | 0.000 | | | | |
| **SJ** | 0.286 | 0.203 | 0.198 | 0.289 | 0.137 | 0.173 | 0.257 | 0.376 | 0.048 | 0.000 | | | |
| **GY** | 0.287 | 0.247 | 0.244 | 0.283 | 0.138 | 0.186 | 0.263 | 0.380 | 0.082 | 0.086 | 0.000 | | |
| **PSE** | 0.181 | 0.168 | 0.180 | 0.327 | 0.115 | 0.187 | 0.187 | 0.284 | 0.057 | 0.067 | 0.067 | 0.000 | |
| **RM** | 0.206 | 0.199 | 0.212 | 0.327 | 0.152 | 0.187 | 0.219 | 0.322 | 0.078 | 0.086 | 0.121 | 0.053 | 0.000 |

Population geographic distance matrix (km)

| | FJ | SE | CF | SC | VJ | AO | PS | EH | LP | SJ | GY | PSE | RM |
|---|---|---|---|---|---|---|---|---|---|---|---|---|---|
| **FJ** | 0.00 | | | | | | | | | | | | |
| **SE** | 7.07 | 0.00 | | | | | | | | | | | |
| **CF** | 3.46 | 4.58 | 0.00 | | | | | | | | | | |
| **SC** | 25.27 | 31.58 | 28.73 | 0.00 | | | | | | | | | |
| **VJ** | 23.83 | 30.18 | 27.28 | 1.46 | 0.00 | | | | | | | | |
| **AO** | 24.21 | 30.50 | 27.67 | 1.10 | 0.64 | 0.00 | | | | | | | |
| **PS** | 7.78 | 4.90 | 7.43 | 29.06 | 27.72 | 27.96 | 0.00 | | | | | | |
| **EH** | 13.52 | 10.17 | 13.39 | 31.11 | 29.91 | 30.03 | 5.97 | 0.00 | | | | | |
| **LP** | 19.20 | 25.94 | 22.59 | 7.13 | 5.77 | 6.37 | 24.18 | 27.33 | 0.00 | | | | |
| **SJ** | 31.43 | 38.07 | 34.84 | 7.31 | 8.40 | 8.34 | 35.93 | 38.30 | 12.26 | 0.00 | | | |
| **GY** | 48.32 | 55.33 | 51.48 | 26.62 | 27.55 | 27.60 | 53.99 | 57.05 | 29.84 | 19.37 | 0.00 | | |
| **PSE** | 12.06 | 10.15 | 9.01 | 36.44 | 34.98 | 35.44 | 14.95 | 20.26 | 29.76 | 41.90 | 56.98 | 0.00 | |
| **RM** | 17.92 | 11.33 | 15.91 | 39.73 | 38.45 | 38.64 | 11.05 | 9.35 | 35.19 | 46.75 | 65.01 | 18.61 | 0.00 |

Population environmental distance matrix (normalised elevation, vegetation, and climate)

| | FJ | SE | CF | SC | VJ | AO | PS | EH | LP | SJ | GY | PSE | RM |
|---|---|---|---|---|---|---|---|---|---|---|---|---|---|
| **FJ** | 0.000 | | | | | | | | | | | | |
| **SE** | 0.271 | 0.000 | | | | | | | | | | | |
| **CF** | 0.271 | 0.000 | 0.000 | | | | | | | | | | |
| **SC** | 3.952 | 3.799 | 3.799 | 0.000 | | | | | | | | | |
| **VJ** | 3.639 | 3.462 | 3.462 | 0.778 | 0.000 | | | | | | | | |
| **AO** | 4.099 | 3.935 | 3.935 | 0.241 | 0.778 | 0.000 | | | | | | | |
| **PS** | 0.679 | 0.567 | 0.567 | 3.436 | 3.055 | 3.577 | 0.000 | | | | | | |
| **EH** | 0.679 | 0.567 | 0.567 | 3.436 | 3.055 | 3.577 | 0.000 | 0.000 | | | | | |
| **LP** | 1.830 | 1.658 | 1.658 | 2.184 | 1.809 | 2.313 | 1.269 | 1.269 | 0.000 | | | | |
| **SJ** | 2.771 | 2.624 | 2.624 | 1.192 | 1.010 | 1.352 | 2.246 | 2.246 | 1.014 | 0.000 | | | |
| **GY** | 0.978 | 1.077 | 1.077 | 4.868 | 4.494 | 4.999 | 1.490 | 1.490 | 2.704 | 3.690 | 0.000 | | |
| **PSE** | 0.392 | 0.121 | 0.121 | 3.736 | 3.388 | 3.866 | 0.554 | 0.554 | 1.591 | 2.565 | 1.140 | 0.000 | |
| **RM** | 0.151 | 0.121 | 0.121 | 3.866 | 3.539 | 4.006 | 0.604 | 0.604 | 1.731 | 2.687 | 1.025 | 0.241 | 0.000 |

Proportion of male flower cooccurrence matrix (eq. 1, below diagonal) and overlap matrix in proportion of male flowering (eq. 2, above diagonal).

| | FJ | SE | CF | SC | VJ | AO | PS | SJ | GY | RM |
|---|---|---|---|---|---|---|---|---|---|---|
| **FJ** | | 0.266 | 0.132 | 0.099 | 0.296 | 0.419 | 0.031 | 0.081 | 0.100 | 0.598 |
| **SE** | 0.250 | | 0.066 | 0.090 | 0.338 | 0.445 | 0.176 | 0.144 | 0.359 | 0.306 |
| **CF** | 0.095 | 0.129 | | 0.227 | 0.169 | 0.209 | 0.782 | 0.650 | 0.110 | 0.454 |
| **SC** | 0.100 | 0.134 | 0.223 | | 0.749 | 0.663 | 0.480 | 0.189 | 0.408 | 0.230 |
| **VJ** | 0.277 | 0.173 | 0.152 | 0.632 | | 0.851 | 0.270 | 0.207 | 0.096 | 0.198 |
| **AO** | 0.339 | 0.248 | 0.214 | 0.584 | 0.729 | | 0.404 | 0.218 | 0.237 | 0.274 |
| **PS** | 0.050 | 0.191 | 0.581 | 0.398 | 0.190 | 0.286 | | 0.747 | 0.392 | 0.453 |
| **SJ** | 0.100 | 0.144 | 0.503 | 0.128 | 0.114 | 0.125 | 0.617 | | 0.152 | 0.441 |
| **GY** | 0.150 | 0.378 | 0.095 | 0.294 | 0.089 | 0.202 | 0.314 | 0.167 | | 0.513 |
| **RM** | 0.336 | 0.331 | 0.354 | 0.248 | 0.188 | 0.207 | 0.385 | 0.342 | 0.374 | |
